# Tunicamycin-Induced Endoplasmic Reticulum Stress Damages Complex I in Cardiac Mitochondria

**DOI:** 10.3390/life12081209

**Published:** 2022-08-09

**Authors:** Qun Chen, Jeremy Thompson, Ying Hu, Edward J. Lesnefsky

**Affiliations:** 1 Division of Cardiology, Department of Medicine, Pauley Heart Center, Richmond, VA 23298, USA; 2 Department of Biochemistry and Molecular Biology, Virginia Commonwealth University, Richmond, VA 23298, USA; 3 McGuire Department of Veterans Affairs Medical Center, Medical Service, Richmond, VA 23249, USA

**Keywords:** calpain, oxidative phosphorylation, NDUFS7, NBUPL

## Abstract

Background: Induction of acute ER (endoplasmic reticulum) stress using thapsigargin contributes to complex I damage in mouse hearts. Thapsigargin impairs complex I by increasing mitochondrial calcium through inhibition of Ca^2+^-ATPase in the ER. Tunicamycin (TUNI) is used to induce ER stress by inhibiting protein folding. We asked if TUNI-induced ER stress led to complex I damage. Methods: TUNI (0.4 mg/kg) was used to induce ER stress in C57BL/6 mice. Cardiac mitochondria were isolated after 24 or 72 h following TUNI treatment for mitochondrial functional analysis. Results: ER stress was only increased in mice following 72 h of TUNI treatment. TUNI treatment decreased oxidative phosphorylation with complex I substrates compared to vehicle with a decrease in complex I activity. The contents of complex I subunits including NBUPL and NDUFS7 were decreased in TUNI-treated mice. TUNI treatment activated both cytosolic and mitochondrial calpain 1. Our results indicate that TUNI-induced ER stress damages complex I through degradation of its subunits including NDUFS7. Conclusion: Induction of the ER stress using TUNI contributes to complex I damage by activating calpain 1.

## Highlights:

- Interfering with protein synthesis increases ER stress and subsequent calpain 1 activation
- ER stress mainly damages the mitochondrial respiratory chain at complex I
- ER stress induces complex I damage by degrading subunits, likely through activation of mitochondrial calpain 1
- Attenuation of the ER stress is a potential approach to improve mitochondrial function in chronic cardiac conditions such as aging and heart failure that have increased ER stress.

## 1. Introduction

The endoplasmic reticulum (ER) contributes to a critical role in protein folding, lipid synthesis, and calcium homeostasis [1]. Since the ER is connected with mitochondria through mitochondria associated membranes [2], ER stress directly affects mitochondrial function [2]. Induction of acute ER stress using thapsigargin impairs mitochondrial function in mouse and rat hearts [3,4,5]. An increase in ER stress with doxorubicin or bevacizumab treatment also results in mitochondrial dysfunction [6,7]. Mitochondrial dysfunction contributes to cardiac injury in many pathological conditions including aging and heart failure [8]. An impaired mitochondrial electron transport chain increases cardiac injury by augmenting the generation of reactive oxygen species (ROS) and sensitizing to mitochondrial permeability transition pore (MPTP) opening [8,9]. Interestingly, inhibition of mitochondrial respiration using antimycin A [10] or rotenone [11] also increases the ER stress. Thus, the damaged mitochondria can further increase the ER stress to worsen the mitochondrial damage during pathologic conditions including aging.

The ER is a key site of intracellular calcium storage in that a greater calcium concentration is required for proper folding of proteins in the ER lumen [12]. Thapsigargin is a Ca^2+^-ATPase inhibitor that blocks calcium reuptake into the ER. Thus, thapsigargin is used to induce ER stress by lowering calcium in the ER lumen [3,4,5]. Blockade of calcium reuptake into the ER by thapsigargin leads to increased cytosolic and mitochondrial calcium [13]. Calpains are a family of calcium-dependent cysteine proteases. Calpain 1 (CPN1) is a ubiquitous calpain isoform located in both cytosol and mitochondria [14,15,16]. Activation of cytosolic CPN1 increases cell injury during cardiac ischemia-reperfusion [15,17,18]. Activation of mitochondria-localized CPN1 (mCPN1) contributes to mitochondrial damage during ischemia and reperfusion [19,20,21,22,23], diabetic cardiomyopathy [24], doxorubicin-induced cardiotoxicity [25,26], and heart failure [27]. Our previous study found that the induction of ER stress using thapsigargin led to mitochondrial dysfunction [13]. Genetic deletion of CPN1 attenuates mitochondrial damage in thapsigargin-treated hearts, supporting that activation of mCPN1 contributes to mitochondrial dysfunction during ER stress [13].

ER stress occurs in many pathological conditions including aging [28,29,30], diabetic disease [24], doxorubicin cardiotoxicity [31], and heart failure [27]. Attenuation of the ER stress leads to improved mitochondrial function in aged hearts, indicating that the ER stress contributes to mitochondrial dysfunction during aging [28,29,30]. However, the mechanism by which aging leads to increased ER stress is unknown. An increase in protein misfolding is a common factor to induce the ER stress [5]. Thus, tunicamycin (TUNI) is often used to induce ER stress by increasing protein misfolding through direct interference with protein glycosylation [32,33]. We asked if the induction of ER stress using TUNI can cause mitochondrial dysfunction in adult mice. We also studied if TUNI-induced mitochondrial dysfunction is dependent on CPN1 activation.

## 2. Methods and Materials

### 2.1. Induction of ER Stress in C57BL/6 Mice Using Tunicamycin

Adult C57BL/6 male mice were purchased from Jackson Laboratories (Bar Harbor, ME, USA). TUNI (0.4 mg/kg) was administered to C57BL/6 mice in vivo through a one-time intraperitoneal (i.p.) injection. TUNI was first dissolved in DMSO and diluted with saline for injection [5]. DMSO and saline solution were used as vehicle treatment. After 24- or 72-h of TUNI or vehicle treatment, mice were anesthetized with pentobarbital sodium (100 mg/kg, i.p.,) and the heart was harvested for mitochondrial isolation [14].

### 2.2. Isolation of Cytosol and Mitochondria from a Single Mouse Heart

Heart mitochondria were isolated as previously described [34]. The harvested mouse heart was quickly blotted dry, weighed, and minced in cold buffer A (composition in mM: 100 KCl, 50 MOPS [3-(N-morpholino) propanesulfonic acid], 1 EGTA, 5 MgSO_4_, and 1 ATP). The minced heart tissue was homogenized using a polytron tissue homogenizer at 10,000 rpm for 2.5 s in the presence of trypsin (5 mg/g tissue). Trypsin was used to increase mitochondrial protein yield and remove potential cytosolic contamination. The homogenate was incubated with trypsin for 15 min at 4 °C, and then diluted with the same volume of buffer B [buffer A + 0.2% bovine serum albumin (BSA)]. The mixture was centrifuged at 500× *g* for 10 min. The supernatant was further centrifuged at 3000× *g* to pellet mitochondria. The mitochondrial pellet was washed with KME buffer (100 mM KCl, 50 mM MOPS, 0.5 mM EGTA), and centrifuged at 3000× *g* to yield the final mitochondrial pellet. Mitochondria were re-suspended in KME for functional study [35].

### 2.3. Mitochondrial Oxidative Phosphorylation, Enzyme Activity, and H_2_O_2_ Generation

The rate of oxygen consumption in mitochondria was measured using a Clark-type oxygen electrode at 30 °C as previously described [36]. Mitochondria were incubated in oxidative phosphorylation buffer (composition in mM: 80 KCl, 50 MOPS, 1 EGTA, 5 KH_2_PO_4_, and 1 mg defatted, dialyzed bovine serum albumin/mL at pH 7.4). Glutamate (20 mM) + malate (10 mM) or pyruvate (20 mM) + malate (10 mM) were used as complex I substrate. Succinate (20 mM) + rotenone (7.5 μM) was used as complex II substrate [37]. TMPD (N,N,N,N′-tetramethyl-p-phenylenediamine)-ascorbate + rotenone (7.5 μM) was used as complex IV substrate. Enzyme activities of the ETC were determined in detergent-solubilized frozen-thawed mitochondria with previously published methods [37]. H_2_O_2_ generation was determined in freeze-thawing mitochondria based on our recently published method [38]. The amount of H_2_O_2_ from mitochondria was measured using the oxidation of the fluorogenic indicator amplex red in the presence of horseradish peroxidase (HRP) [39]. Frozen mitochondria were solubilized in 5% cholate, and NADH (1 μM) was used as substrate [39].

### 2.4. Immunoblotting

Cytosol or mitochondrial samples were solubilized in sample buffer and denatured at 95 °C for 5 min. Samples were separated using 12% or 4–15% tris-glycine gels (Bio-Rad, Hercules, CA, USA) and transferred to PVDF membrane by semi-dry transfer (Bio-Rad). The membranes were incubated for 1 h at room temperature in 5% (*w*/*v*) non-fat dry milk (Bio-Rad) in TBST buffer (10 mM Tris pH 7.5, 150 mM NaCl, 0.1% Tween20). The membrane was washed with TBST for 5 min at room temperature. Then, the membrane was incubated with primary antibodies at 4 °C overnight (See information in Table 1). The membrane was washed with TBST buffer before addition of secondary antibody (HRP-conjugated anti-mouse or anti-rabbit IgG F(ab)_2_, 1:10,000 dilution, GE Healthcare Life Sciences, Piscataway, NJ, USA) and incubated for 1 h in room temperature. The blots were developed using ECL Plus Western Blotting Detection Reagents (GE Healthcare Life Sciences, Piscataway, NJ, USA). Membranes were digitally analyzed (Bio-Rad, Hercules, CA, USA) using Image Lab 6.0 software.

### 2.5. Measurement of Cardiac Function Using Echocardiography

Echocardiography and doppler echocardiography were performed using the Vevo770^TM^ imaging system (VisualSonics Inc., Toronto, ON, Canada) in the mice with or without TUNI treatment. Mice were anesthetized with isoflurane and placed in the supine position. The chest was carefully shaved, and ultrasound gel applied to the thorax to optimize visibility during the exam. Echocardiography was used to quantify LV chamber diameter, wall thickness, fractional shortening, ejection fraction, stroke volume, and cardiac output.

### 2.6. Statistical Analyses

Data were expressed as the mean ± standard error. Differences between groups (≥3 groups) were compared by one-way ANOVA when data passed normality and equal variance tests. When a significant F value was obtained, means were compared using the Student–Newman–Keuls test of multiple comparisons. Differences between two groups were compared by unpaired student *t*-test (SigmaStat 3.5, Systat, Richmond, CA, USA). Statistical significance was defined as a value of *p* < 0.05.

## 3. Results

### 3.1. Chronic TUNI Treatment Increased ER Stress

**Figure 1:**
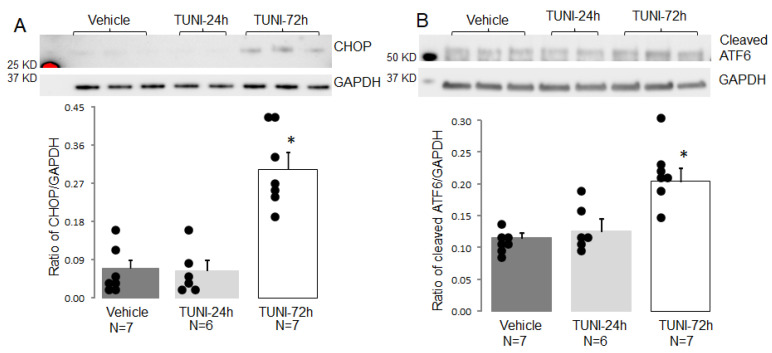
TUNI treatment increased ER stress in C57BL/6 mice. Panel (**A**) showed that 72 h TUNI treatment increased CHOP (C/EBP homologous protein) expression compared to vehicle or 24 h TUNI. Panel (**B**) showed that 72 h TUNI treatment increased ATF6 (activating transcription factor 6) cleavage compared to vehicle or 24 h TUNI. Mean ± SEM, * *p* < 0.05 vs. vehicle or 24 h TUNI treatment.

An increase in the contents of CHOP (C/EBP homologous protein) and cleaved ATF6 (activating transcription factor 6) is used as an indicator of ER stress. Thus, CHOP and cleaved ATF6 were measured in cytosol isolated from vehicle- and TUNI-treated mouse hearts. TUNI treatment for 24 h did not increase the contents of CHOP nor cleaved ATF6 compared to vehicle (Figure 1A,B). However, TUNI treatment for 72 h significantly increased the contents of CHOP and cleaved ATF6 compared to vehicle (Figure 1A,B). These results indicate that chronic TUNI treatment increases the ER stress in mouse heart.

### 3.2. Chronic TUNI Treatment Decreased Oxidative Phosphorylation in Cardiac Mitochondria

**Figure 2:**
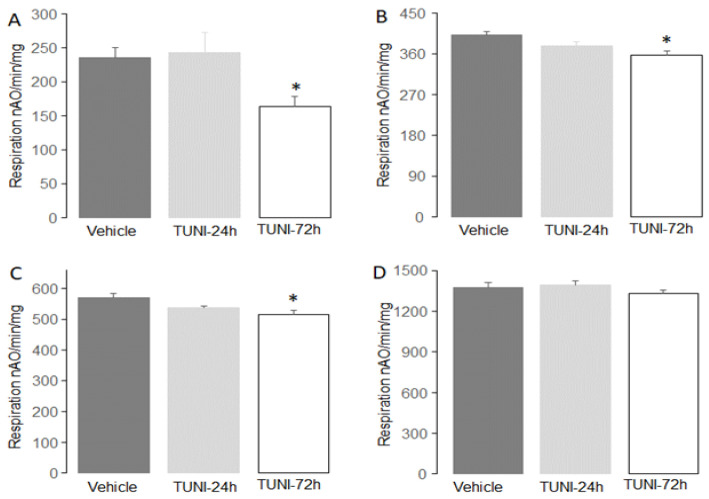
TUNI treatment led to decreased oxidative phosphorylation in C57BL/6 mice. Panel (**A**) shows that 72 h following TUNI treatment oxidative phosphorylation was decreased using glutamate + malate as complex I substrates. TUNI treatment also decreased oxidative phosphorylation using pyruvate + malate (Panel (**B**)) or succinate (Panel (**C**)) as substrates. Panel (**D**) shows that 72 h following TUNI treatment did not alter oxidative phosphorylation using TMPD-ascorbate as complex IV substrate. Mean ± SEM, * *p* < 0.05 vs. vehicle or 24 h TUNI treatment. N = 6–10 in each group.

The rate of oxidative phosphorylation was measured in cardiac mitochondria isolated from mice with or without TUNI treatment. There were no differences in oxidative phosphorylation between vehicle and 24 h TUNI treatment using complex I (Figure 2A,B), complex II (Figure 2C), or complex IV substrates (Figure 2D). Compared to vehicle, 72 h following TUNI treatment led to decreased oxidative phosphorylation in mitochondria using glutamate + malate (Figure 2A) and pyruvate + malate as complex I substrates. Succinate oxidation was also slightly decreased in mitochondria from mice 72 h following TUNI treatment compared to vehicle (Figure 2C). TUNI treatment did not alter TMPD-ascorbate oxidation even after 72 h (Figure 2D). These results show that chronic TUNI treatment mainly decreases oxidative phosphorylation in mitochondria oxidizing complex I substrates.

### 3.3. Chronic TUNI Treatment Decreased Complex I Activity in Cardiac Mitochondria

**Figure 3:**
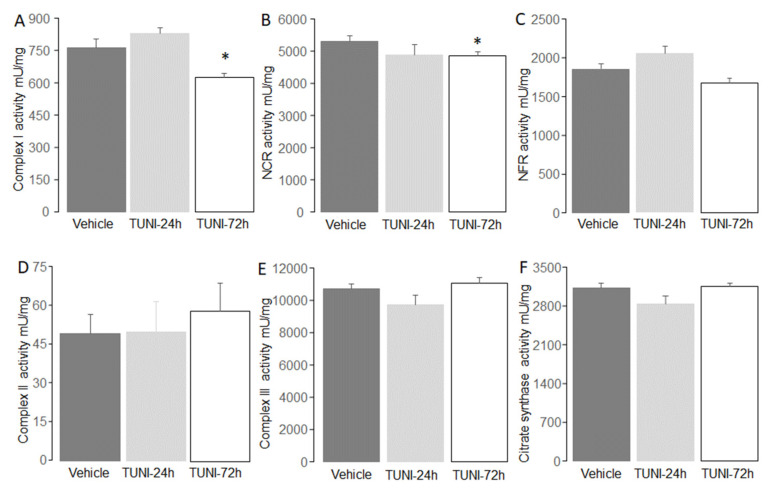
TUNI treatment decreased complex I activity in C57BL/6 mice. Panel (**A**) showed that 72 h TUNI treatment decreased complex I activity compared to vehicle or 24 h TUNI. TUNI treatment also decreased the activity of NCR (Panel (**B**)). However, NFR activity was not altered in TUNI-treated mice (Panel (**C**)). TUNI treatment did not alter the activities of complex II (Panel (**D**)), complex III (Panel (**E**)), nor citrate synthase (Panel (**F**)). Mean ± SEM, * *p* < 0.05 vs. vehicle or 24 h TUNI treatment. N = 6–10 in each group.

Since TUNI treatment for 24 h did not alter oxidative phosphorylation, enzyme activity was measured in mitochondria 72 h following TUNI or vehicle treatment. Compared to vehicle, complex I activity was decreased in mitochondria following 72 h (Figure 3A). TUNI treatment also decreased NCR (NADH:cytochrome *c* oxidoreductase) activity compared to vehicle (Figure 3B). TUNI treatment did not alter the activities of NFR (NADH:ferricyanide oxidoreductase) (Figure 3C), complex II (Figure 3D), complex III (Figure 3E), and citrate synthase (Figure 3F) compared to vehicle. These results support that TUNI treatment leads to complex I damage.

### 3.4. TUNI Treatment Led to Decreased Contents of Complex I Subunits in Cardiac Mitochondria 72 h Following Exposure

**Figure 4:**
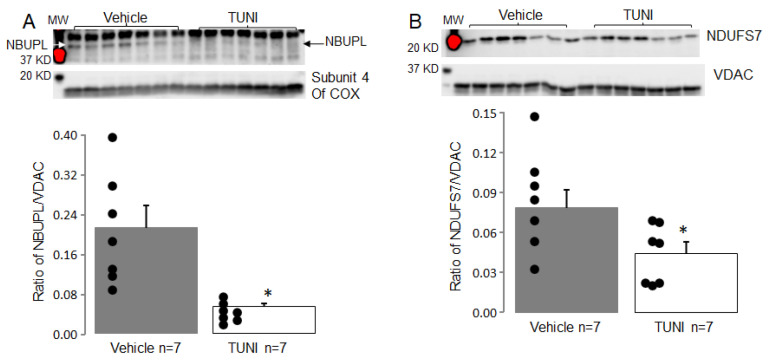
TUNI treatment decreased contents of complex I subunits in C57BL/6 mice. The 72 h of TUNI treatment led to decreased contents of NBUPL (Panel (**A**)) and NDUFS7 (Panel (**B**)) compared to vehicle or 24 h TUNI. Mean ± SEM, * *p* < 0.05 vs. vehicle or 24 h TUNI treatment.

**Figure 5:**
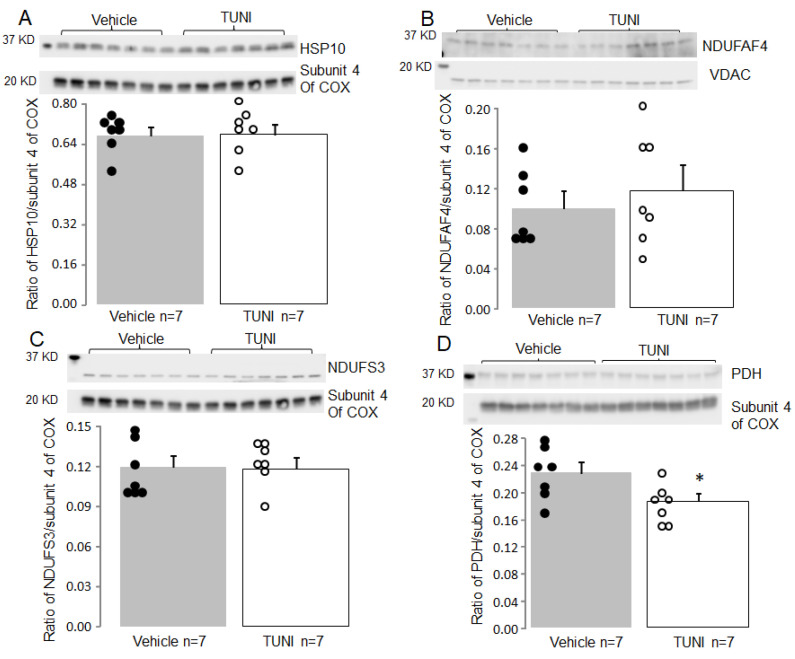
Effect of TUNI treatment on the contents of complex I and PDH subunits in C57BL/6 mice. 72 h of TUNI treatment did not alter HSP10 (Panel (**A**)), NDUFAF4 (Panel (**B**)), and NDUFS3 (Panel (**C**)). HSP10 and NDUFS3 were run on the same gel with subunit 4 of cytochrome oxidase (COX) as loading control. TUNI treatment decreased the content of PDH α1 subunit (Panel (**D**)) compared to vehicle treatment. Mean ± SEM, * *p* < 0.05 vs. vehicle or 24 h TUNI treatment.

Immunoblotting was used to assess the contents of selected complex I subunits. TUNI treatment led to decreased contents of NBUPL (Figure 4A) and NDUFS7 (Figure 4B) compared to vehicle. TUNI treatment did not alter the contents of HSP10 (Figure 5A), NDUFAF4 (Figure 5B), and NDUFS3 (Figure 5C) compared to vehicle. These results suggest that TUNI treatment decreases complex I activity by degrading specific subunits 72 h after treatment.

### 3.5. TUNI Treatment Led to the Degradation of a PDH Subunit in Cardiac Mitochondria

TUNI treatment led to decreased pyruvate oxidation (Figure 2B). Thus, the content of PDH α1 subunit was measured in mitochondria isolated from hearts with or without TUNI treatment for 72 h. TUNI treatment decreased the content of the PDH α1 subunit compared to vehicle (Figure 5D). This result suggests that TUNI treatment decreases pyruvate oxidation by impairing PDH.

### 3.6. TUNI Treatment Increased H_2_O_2_ Generation in Cardiac Mitochondria

The total H_2_O_2_ generation was measured in frozen-thawed cardiac mitochondria with NADH as substrate [38]. Compared to vehicle, 72 h of TUNI treatment increased total H_2_O_2_ (pmol/min/mg) generation in cardiac mitochondria [mean ± SEM, vehicle (512 ± 14) vs. TUNI (568 ± 5), *p* < 0.05, *n* = 8 in each group]. These results suggest that TUNI-induced ER stress increases oxidative stress in cardiac mitochondria.

### 3.7. TUNI Treatment Activated Cytosolic and Mitochondrial Calpain 1 (CPN1)

**Figure 6:**
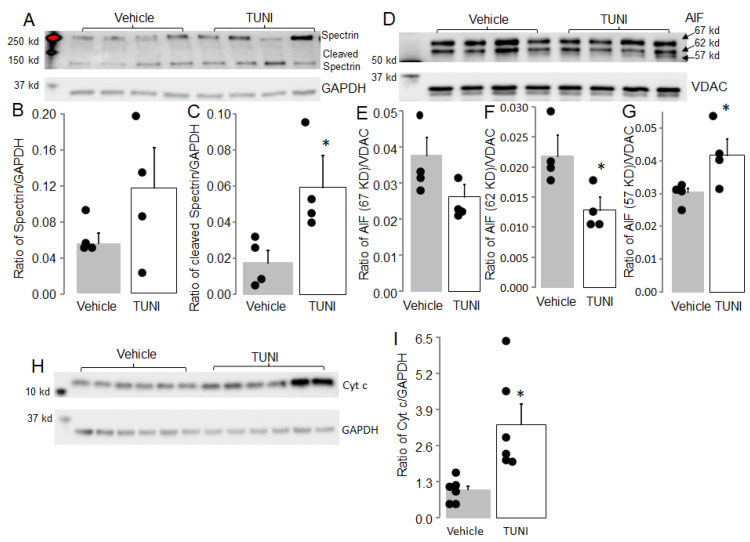
TUNI treatment activated cytosolic and mitochondrial calpain 1. Seventy two hours after TUNI treatment, the cleavage of spectrin, a substrate of cytosolic calpain 1, was increased (Panel (**A**,**C**)). However, TUNI treatment did not alter total spectrin content compared to vehicle (Panel (**A**,**B**)). TUNI treatment did not alter content of AIF (67 kd) compared to vehicle treatment (Panel (**D**,**E**)). TUNI-activated mitochondrial calpain 1, as shown, increased AIF truncation (Panel (**D**,**F**,**G**)). TUNI treatment also increased the release of cytochrome *c* into cytosol compared to vehicle (Panel (**H**,**I**)). Mean ± SEM, * *p* < 0.05 vs. vehicle or 24 h TUNI treatment. N = 4–6 in each group.

Activation of CPN1 contributes to mitochondrial dysfunction during ischemia-reperfusion [38] and aging [30]. Spectrin is a substrate of cytosolic CPN1. A decrease in spectrin content or an increase in cleaved spectrin indicates cytosolic CPN1 activation. Compared to vehicle, TUNI treatment did not alter spectrin content (Figure 6A,B). However, the content of cleaved spectrin was increased in TUNI treated mice compared to vehicle (Figure 6A,C). This result indicates that TUNI treatment activates cytosolic CPN1.

AIF is a substrate of mitochondrial CPN1. AIF is a nuclear-encoded protein that is imported into mitochondria as a 67 kd precursor. Mature AIF (62 kd) is formed after removal of the mitochondrial leader sequence. Activation of CPN1 cleaves AIF to form truncated AIF (57 kd). Thus, a decrease in 62 kd AIF or an increase in 57 kd AIF indicates mitochondrial CPN1 activation. TUNI treatment did not alter the 67 kd AIF content compared to vehicle (Figure 6D,E). However, TUNI treatment led to decreased 62 kd AIF and increased 57 kd AIF compared to vehicle (Figure 6D,F,G). These results support that mitochondrial CPN1 is activated 72 h following TUNI treatment.

### 3.8. TUNI Treatment Increased Cytochrome c Release into Cytosol

A release of cytochrome *c* from mitochondria into cytosol is a critical step to trigger apoptosis. The content of cytochrome *c* in cytosol in TUNI-treated mice was significantly greater than that in vehicle-treated mice (Figure 6H,I). The result supports that TUNI treatment leads to a release of cytochrome *c* from mitochondria into cytosol.

### 3.9. TUNI Treatment Had Limited Effect on Cardiac Function

**Figure 7:**
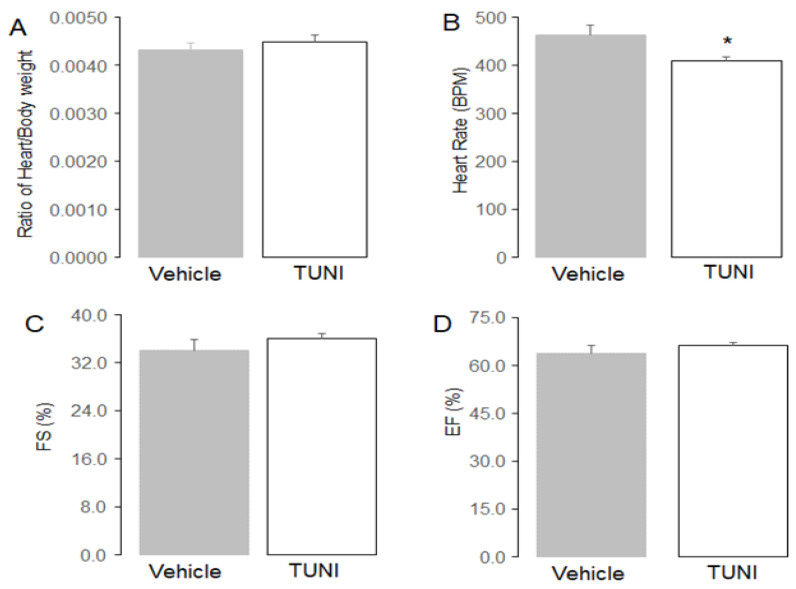
The effect of TUNI treatment on cardiac function in C57BL/6 mice. Following 72 h, TUNI treatment did not alter the ratio of heart/body weight (Panel (**A**)). TUNI treatment led to decreased heart rate (Panel (**B**)). However, TUNI treatment did not alter fractional shortening (FS) (Panel (**C**)) nor ejection fraction (EF) (Panel (**D**)) compared to vehicle. Mean ± SEM, * *p* < 0.05 vs. vehicle. N = 5 in each group.

Cardiac function was measured in separate groups of mice with or without TUNI treatment. TUNI treatment did not alter the ratio of heart/body weight (Figure 7A). Compared to vehicle, TUNI treatment led to decreased heart rate (Figure 7B). However, TUNI treatment did not alter ejection fraction (Figure 7D) nor the left ventricular fractional shortening, a second index of contractile function (Figure 7C). These results indicate that TUNI treatment has limited effect on cardiac function 72 h following treatment.

## 4. Discussion

Our previous study found that the induction of ER stress using thapsigargin leads to decreased complex I activity in cardiac mitochondria through degradation of complex I subunits [13]. In the present study, we find that the induction of ER stress using TUNI also damages complex I in cardiac mitochondria. TUNI treatment activates both cytosolic and mitochondrial CPN1. Thus, activation of mitochondrial CPN1 likely contributes to the complex I damage during TUNI-induced ER stress. These results indicate that complex I is a downstream target in mitochondria following the induction of ER stress. Activation of mitochondria-localized CPN1 may contribute to mitochondrial and complex I damage during ER stress.

### 4.1. Induction of the ER Stress with Thapsigargin and TUNI

The ER is a network of membranes that function for post-translational processing of proteins including proper protein folding [1,40]. The accumulation of misfolded proteins within the ER triggers ER stress [5,41]. Although the initial ER stress is an adaptive reaction attempting to restore the ER function by slowing protein synthesis and folding, severe ER stress leads to cell injury and death [1,3,5]. An oxidizing environment and high Ca^2+^ content in the ER lumen are required for Ca^2+^-dependent chaperones to stabilize protein folding through the formation of disulfide bonds [42]. Thus, the reduction of Ca^2+^ inside the ER as a result of thapsigargin exposure induces the ER stress by decreasing calcium uptake into the ER through inhibition of Ca^2+^-ATPase [5,43]. In contrast, TUNI induces ER stress by increasing protein misfolding through direct interference with protein glycosylation [32,33]. Thus, thapsigargin and TUNI induce ER stress via different mechanisms.

Since thapsigargin blocks calcium uptake into the ER, the cytosolic and mitochondrial calcium level is quickly elevated that favors calcium-dependent CPN1 activation [13]. ER stress is increased in mice within 72 h of TUNI treatment. Twenty four hours of TUNI treatment does not induce appreciable ER stress and potential CPN1 activation. These results indicate that TUNI treatment leads to slowly increased ER stress. Interestingly, mitochondrial dysfunction only occurs in mice at the 72 h assessment point following TUNI treatment, indicating that TUNI-induced ER stress contributes to the mitochondrial dysfunction. Thus, the induction of the ER stress using thapsigargin [13] or TUNI leads to mitochondrial dysfunction.

### 4.2. ER Stress and Complex I Damage

Mitochondrial dysfunction contributes to cardiac injury in pathological conditions including aging, heart failure, and ischemia-reperfusion [8,9]. Complex I is a major site of dysfunction in the electron transport chain in aged mouse heart mitochondria [28,29]. The damaged complex I augments cardiac injury by increasing ROS generation [44] and the probability of mitochondrial permeability transition opening [45]. Improvement of mitochondrial function and complex I activity leads to decreased cardiac injury in aged hearts following ischemia-reperfusion [28,29,30], supporting that age-induced complex I defects plays a key role in cell injury during superimposed disease stress in aging mice [30].

Complex I is an L-shaped molecular complex that includes a membrane arm embedded in the mitochondrial inner membrane and a second peripheral arm extending into the mitochondrial matrix [46]. The peripheral arm contains 7 core subunits that function in NADH oxidation and subsequent electron transfer through complex I [46]. Subunits in the membrane arm are responsible for coupled proton translocation across the inner membrane [46]. Thapsigargin treatment leads to decreased complex I activity through the degradation of NDUFB1 (NADH:ubiquinone oxidoreductase subunit B1) and NDUFS7 (NADH:Ubiquinone oxidoreductase core subunit S7). NDUFB1 is one of the hydrophobic subunits of complex I that functions in anchoring the complex I at the inner membrane. An NDUFB1 defect impairs complex I in human skeletal muscle dysferlinopathy [47]. NDUFS7 is one of the core subunits functioning in electron transfer to ubiquinone [48]. Mutation of the NDUFS7 gene contributes to a complex I defect observed in some Leigh syndrome patients [49]. NDUFS7 deficiency is involved in complex I damage during thapsigargin-induced ER stress [13]. In the current study, we find that NDUFS7 content is decreased in TUNI-treated hearts. The result suggests that the NDUFS7 defect plays an essential role in ER stress-mediated complex I damage. In addition, we find that TUNI leads to decreased NBUPL (nucleotide-binding protein-like protein) content. NBUPL has an essential role in the assembly of complex I [46]. This result suggests that ER stress also leads to decreased complex I activity by potentially impairing assembly of the intact complex.

Aging impairs complex I by decreasing the contents of complex I subunits including NDUFAF4 and HSP10 [28]. Since ER stress is involved in the complex I defect during aging, we studied the impact of TUNI treatment on NDUFAF4 and HSP10. Surprisingly, TUNI treatment did not alter the contents of NDUFAF4 nor HSP10. These results suggest that ER stress is not the sole factor to induce the complex I defect during aging. Ischemia-reperfusion impairs complex I by decreasing NDUFS3 content [50]. Thus, we also studied NDUFS3 in TUNI-treated mitochondria. TUNI treatment also did not alter NDUFS3 content. These results indicate that complex I defects in different cardiac disease states can be induced through different mechanisms that lead to the decrease in content of specific subunits.

### 4.3. ER Stress and CPN1 Activation

The activation of CPN1 contributes to mitochondrial damage during ischemia-reperfusion [14,23,51,52]. A recent study shows that activation of mitochondrial CPN1 impairs complex I function in aged heart mitochondria [30]. The activation of CPN1 can also increase the ER stress in cardiac myocytes [53]. The induction of ER stress using thapsigargin also activates CPN1 [13]. Interestingly, we found that TUNI treatment also activated cytosolic and mitochondrial CPN1. NDUFS7 is a substrate of CPN1 [54]. Both thapsigargin and TUNI treatment led to decreased NDUFS7 content accompanied by activated mitochondrial CPN1, suggesting that CPN1-mediated NDUFS7 degradation contributes to a critical role in the ER stress-mediated complex I damage.

Since thapsigargin treatment increases mitochondrial calcium concentration, it is easy to make a connection between thapsigargin-induced ER stress and CPN1 activation. In contrast, TUNI treatment increases ER stress by interfering with protein folding. In other words, since TUNI treatment does not directly increase mitochondrial calcium, it is less clear how TUNI treatment leads to CPN1 activation. ER stress impairs mitochondrial function that increases oxidative stress [28]. Oxidative stress can increase calcium overload in cardiac myocytes by decreasing Ca^2+^-ATPase activity through prevention of ATP binding to the Ca^2+^-ATPase [55,56,57]. ER stress also increases oxidative stress [58] that can decrease the threshold of calcium to activate CPN1 [59]. An increase in total H_2_O_2_ generation in TUNI-treated mitochondria supports the increased oxidative stress in the current study. Thus, TUNI treatment can activate CPN1 by increasing intracellular and mitochondrial calcium content indirectly via the potential induction of oxidative stress leading to calcium overload indirectly through the inhibition of the Ca^2+^-ATPase.

The pyruvate dehydrogenase (PDH) α1 subunit is another CPN1 substrate [52]. TUNI treatment leads to decreased pyruvate oxidation. The content of the PDHα1 subunit is also decreased in mitochondria from TUNI-treated hearts. These results indicate that ER stress impairs pyruvate oxidation likely by decreasing PDH activity through degradation of the α1 subunit. The results also further support that TUNI-induced ER stress leads to mitochondrial CPN1 activation. PDHα1 is a potential calpain target [4]. ER stress also leads to decreased PDH α1 subunit content in cardiac mitochondria [4]. Incubation of cardiac mitochondria with exogenous calcium leads to decreased pyruvate oxidation, whereas genetic inhibition of CPN1 protects pyruvate oxidation in calcium-treated mitochondria, supporting that activation of CPN1 contributes to PDH subunit degradation [13].

In addition to calcium-dependent protease activation, disruption of calcium interplay between ER and mitochondria can also impair mitochondrial function [60]. Although calcium overload is detrimental to mitochondria, a proper calcium level within the mitochondrial matrix is also required to maintain normal mitochondrial function. Induction of the ER stress using TUNI can lead to calcium deletion inside the ER that disrupts calcium communication between the ER and mitochondria [60]. Modulation of calcium communication between ER and mitochondria using sigma-1 receptor activators improves mitochondrial function [61,62]. Thus, TUNI treatment may impair mitochondrial function through interruption of calcium signaling between ER and mitochondria.

### 4.4. ER Stress and Cardiac Dysfunction

Aging leads to alterations in cardiac geometry and function due to cardiac hypertrophy, inflammation, and fibrosis. Echocardiographic evaluation shows that aging mainly affects the diastolic function in male C57BL/6 mice [63]. Aging also leads to decreased contractility in isolated cardiomyocytes [64]. These results indicate that aging may affect both systolic and diastolic function. In the present study, we find that 72 h following TUNI treatment, systolic function is preserved. This result indicates that TUNI-induced ER stress only has a mild effect on cardiac function. In contrast, mitochondrial dysfunction already occurs in mice within 72 h of TUNI treatment. The disparity between mitochondrial dysfunction and the mild alteration of cardiac function in TUNI-treated mice suggests that mitochondrial dysfunction is only a trigger of cardiac dysfunction during ER stress. A longer time frame is needed to manifest cardiac dysfunction in TUNI-treated mice by developing cardiomyocyte injury and dysfunction followed by inflammation and fibrosis. However, the current model of TUNI exposure provides an experimental system to study the early events in the mechanism of ER stress-induced cardiac dysfunction. Thus, a more prolonged TUNI treatment period may be needed to potentially recapitulate the aging phonotype.

## 5. Conclusions

The induction of ER stress using thapsigargin or TUNI leads to decreased complex I activity via the decrease in the content of key subunits, likely involving degradation in part through the activation of mitochondrial CPN1. The attenuation of ER stress or the prevention of CPN1 activation is a potential strategy to protect complex I in cardiac diseases that manifest increased ER stress as a consequence of impaired protein folding and processing [42].

## Figures and Tables

**Table 1:** Antibodies used in the current manuscript.

Antibody Name	Company	Catalog Number	Concentration
Subunit 4 of cytochrome oxidase	Cell Signaling	4844	1:10,000
GAPDH (Glyceraldehyde-3-Phosphate Dehydrogenase)	Cell Signaling	5174	1:1000
NDUFA4 (mitochondrial complex associated)	Abcam	ab129752	1:1000
NDUFB1 (NADH:Ubiquinone Oxidoreductase Subunit B1)	Abcam	ab201302	1:1000
NDUFS7 (NADH:Ubiquinone Oxidoreductase Core Subunit S7)	ThermoFisher Scientific	PA5-19343	1:500
PDHα1 subunit (pyruvate dehydrogenase α1 subunit)	Cell Signaling	2784	1:1000
Spectrin	Santa Cruz	csc-46696	1:100
VDAC (Voltage-dependent anion-selective channel)	Abcam	ab14715	1:2500

## Data Availability

Not applicable.
